# Supplementary material for: Learning curve and surgical outcome of robotic assisted colorectal surgery with ERAS program
Source: Sci Rep. 2022 Nov 29;12:20566. doi: 10.1038/s41598-022-24665-w (PMC9709162; doi:10.1038/s41598-022-24665-w)
Supplement: Supplementary file 1 — Supplementary Information 1. [file 41598_2022_24665_MOESM1_ESM.zip › LSC Raw data-Ñ[▒K/141/P_ñw╝╨░O▒Kñσ.pdf]

[redacted]

Pathologic diagnosis:
1. Rectum, low anterior resection --- Status post excision with scar and foreign body type giant cell reaction in lamina propria. No residual tumor seen. The bilateral cut ends and perirectal lymph nodes (0/11) are free of tumor.
2. IMA root, dissection --- Unremarkable fibroadipose tissue and blood vessels. Negative for malignancy.
3. IMV root, dissection --- Unremarkable fibroadipose tissue and blood vessels. Negative for malignancy.
4. Rectum, cut end, distal, resection --- Negative for malignancy.

Ancillary study for diagnosis:
Step cut x 3 and CDX2 IHC stain done for section D, and target cells lost in IHC section.

Prognostic and predictive factor:
(Combined with previous excision specimen: S1015258)
1. Tumor size: 0.8 cm. (in S1015258)
2. Depth of invasion: Submucosa. (in S1015258)
3. Histologic grade: G2: Moderately differentiated. (in S1015258)
4. Angiolymphatic invasion: Not identified.
5. Perineural invasion: Not identified.
6. Tumor deposits (discontinuous extramural extension): Not identified.
7. Macroscopic tumor perforation: Not identified.
8. Surgical Margins:
a) Proximal Margin: Uninvolved by invasive carcinoma.
b) Distal Margin: Uninvolved by invasive carcinoma.
c) Radial or Mesenteric Margin: Uninvolved by invasive carcinoma.
9. Lymph node status: Uninvolved (0/11).
10. Extranodal involvement: Not identified.
11. Tumor regression grading S/P CCRT: No known presurgical therapy.
12. Type of polyp in which invasive carcinoma arose: Not identified.
13. Additional pathologic findings: Not identified.
14. Pathological TNM Stage: pT1N0 (According to the eighth edition, American Joint Committee on Cancer Staging Guidelines for Tumors).
15. TNM descriptors: Not applicable.

Gross description:
The specimen consists of 1) a segment of colon, 16 cm in length and 6.5 cm in proximal cut end circumference, 8 cm in distal circumference, and perirectal soft tissue. A surgical stitch marked the previous tumor excision site, which is 1.5 cm from distal cut end and 13.5 cm from proximal cut end. On cross section, there is no mass lesion found grossly. There are two stitches noted on perirectal soft tissue margin, which labeled as "MRI LN+". On cut, there is no enlarged lymph node found in adjacent soft tissue. The both cut ends are unremarkable grossly. 2) IMA root, a piece of tan yellow soft tissue, 2.5x1.6x1.5 cm. 3) a piece of tan soft tissue, 2.6x1.3x1 cm, labeled as "IMV root". 4) distal cut end, a piece of tan mucosa-covered soft tissue, 2x1.8x1 cm. Representative part for section: A) proximal cut end B) distal cut end C-D) colon near one stitch E) radial margin and soft tissue near two stitches F) all of specimen 2 G-H) specimen 3-4. Blue ink indicated serosa; yellow ink indicated radial margin.
#T-59600_2 #M-78060_2 0 1105 SP0000
#T-C4560_2 #M-09183_2 0 1105 000000

已電子簽章

敬告：本單僅供醫師參考使用，不具任何申請效力！！
請病患妥為保管，若不慎遺失需自負個人資料外洩責任！
